# Supplementary material for: Simultaneous Analysis of Free/Combined Phytosterols in Rapeseed and Their Dynamic Changes during Microwave Pretreatment and Oil Processing
Source: Foods. 2022 Oct 14;11(20):3219. doi: 10.3390/foods11203219 (PMC9601674; doi:10.3390/foods11203219)
Supplement: Supplementary file 1 [file foods-11-03219-s001.zip › foods-1923719-supplementary.pdf]

Supporting Information

Tables S1–S5

Table S1. Optimization of the liquid-liquid extraction method/mg/kg.

Extraction method	Combined brassicasterol	Combined campesterol	Combined β-sitosterol	Free brassicasterol	Free campesterol	Free β-sitosterol	Total combined phytosterols	Total free phytosterols	Total phytosterols
Folch	171.23±2.79	633.73±4.04	864.33±4.05	339.56±0.67	458.43±1.08	722.92±1.50	1669.30	1520.91	3190.21
Bligh-Dyer	158.02±1.78	586.87±0.93	798.43±1.35	335.27±0.95	454.93±0.56	711.74±0.31	1543.32	1501.93	3045.26
MTBE	135.54±3.97	519.27±4.17	699.81±4.76	297.29±3.39	405.64±4.25	635.31±4.29	1354.61	1338.23	2692.84

Table S2. Effects of microwave pretreatment on the composition and content of phytosterols in rapeseed/mg/kg.

Rapeseed	Microwave pretreatment	Combined brassicasterol	Combined campesterol	Combined β-sitosterol	Free brassicasterol	Free campesterol	Free β-sitosterol	Total combined phytosterols	Total free phytosterols	Total phytosterols
Zhongyouza 19	without microwave pretreatment	153.96±7.09	562.31±7.48	920.77±8.89	319.30±1.02	421.45±4.02	742.92±3.49	1637.05	1483.67	3120.71
	with microwave pretreatment	158.37±1.10	585.20±3.34	941.75±4.64	316.56±1.59	451.82±1.73	776.54±1.72	1685.31	1544.92	3230.23
Dadi 199	without microwave pretreatment	145.32±2.31	582.49±1.20	833.51±2.14	342.82±2.53	514.63±2.93	799.82±2.08	1561.33	1657.27	3218.61
	with microwave pretreatment	144.46±6.17	573.05±3.69	812.84±4.97	360.08±2.39	546.83±2.28	835.59±2.77	1530.35	1742.49	3272.84

Table S3. Effects of microwave pretreatment on the composition and content of phytosterols in rapeseed oil/mg/kg.

Rapeseed	Microwave pretreatment	Combined brassicasterol	Combined campesterol	Combined β-sitosterol	Free brassicasterol	Free campesterol	Free β-sitosterol	Total combined phytosterols	Total free phytosterols	Total phytosterols
Zhongyouza 19	without microwave pretreatment	267.58±3.23	1113.97±2.64	1704.62±1.86	566.16±1.31	796.67±1.28	1320.69±1.24	3086.17	2683.52	5769.68
	with microwave pretreatment	294.69±0.82	1197.32±0.92	1842.13±0.75	618.59±1.78	872.41±1.90	1441.42±1.96	3334.14	2932.42	6266.56
Dadi 199	without microwave pretreatment	238.28±4.18	1083.27±2.11	1469.52±2.30	599.11±0.44	918.21±0.69	1345.54±1.04	2791.07	2862.86	5653.93
	with microwave pretreatment	240.73±3.93	1095.79±0.88	1466.96±1.20	620.02±1.30	951.47±1.49	1384.87±1.63	2803.48	2956.36	5759.83

Table S4. Effects of microwave pretreatment on the composition and content of phytosterols in rapeseed cake/mg/kg.

Rapeseed	Microwave pretreatment	Combined brassicasterol	Combined campesterol	Combined β-sitosterol	Free brassicasterol	Free campesterol	Free β-sitosterol	Total combined phytosterols	Total free phytosterols	Total phytosterols
Zhongyouza 19	without microwave pretreatment	82.36±1.90	211.97±2.40	378.31±2.41	141.74±0.35	204.98±0.32	386.34±0.50	672.64	733.06	1405.69
	with microwave pretreatment	83.55±1.85	215.70±1.19	388.94±1.11	134.27±0.64	193.58±0.22	377.01±0.36	688.19	704.86	1393.05
Dadi 199	without microwave pretreatment	87.80±3.49	307.20±0.96	452.07±1.34	177.99±1.42	264.97±1.17	432.67±0.26	847.07	875.63	1722.70
	with microwave pretreatment	95.94±2.00	341.26±2.07	516.82±2.67	178.67±0.77	266.50±0.58	455.94±0.57	954.02	901.10	1855.13

Table S5. Effect of microwave pretreatment on oil content in rapeseed, residual oil content in rapeseed cake and oil yield in rapeseed oil processing.

Rapeseed	Microwave pretreatment	Oil content in rapeseed	Residual oil content in rapeseed cake	Oil yield of rapeseed
Zhongyouza 19	without microwave pretreatment	40.80%	13.46%	31.59%
	with microwave pretreatment	42.11%	13.00%	33.45%
Dadi 199	without microwave pretreatment	42.56%	20.33%	27.90%
	with microwave pretreatment	44.85%	20.53%	30.60%
